# Supplementary material for: Meta-analysis indicates better climate adaptation and mitigation performance of hybrid engineering-natural coastal defence measures
Source: Nat Commun. 2024 Apr 9;15:2870. doi: 10.1038/s41467-024-46970-w (PMC11004181; doi:10.1038/s41467-024-46970-w)
Supplement: Supplementary file 1 — Supplementary Information [file 41467_2024_46970_MOESM1_ESM.pdf]

Supplementary Materials for

Meta-analysis indicates better climate adaptation and mitigation performance of hybrid engineering-natural coastal defence measures

Author list

Lam Thi Mai Huynh[1,*], Jie Su[2*], Quanli Wang[2], Lindsay C. Stringer[3,4], Adam D. Switzer[5,6] Alexandros Gasparatos[2,7*]

1. Graduate Program in Sustainability Science - Global Leadership Initiative (GPSS-GLI), The University of Tokyo, 5-1-5 Kashiwanoha, Kashiwa City 277- 8563, Japan.
2. Institute for Future Initiatives (IFI), The University of Tokyo, 7-3-1 Hongo, Bunkyo-ku, Tokyo 131-8654, Japan
3. York Environmental Sustainability Institute, University of York, York, YO10 5NG, United Kingdom
4. Department of Environment and Geography, University of York, York, YO10 5NG, United Kingdom
5. Earth Observatory of Singapore, Nanyang Technological University, Singapore, 50 Nanyang Avenue, Singapore 639798.
6. Asian School of the Environment, Nanyang Technological University, Singapore, 50 Nanyang Avenue, Singapore 639798.
7. Institute for the Advanced Study of Sustainability (UNU-IAS), United Nations University, 5-53-Jingumae, Shibuya-ku, Tokyo 150-8925, Japan

The supplementary materials include:

- Supplementary Table 1 to 12
- Supplementary Box 1-2
- Supplementary Figure 1-7

Supplementary Table 1: Coding for meta-data extraction

Variable	Details	Codes	Comments
**General study characteristics: Code by paper**			
Geography	Geographical focus of the reported studies?		Name of country<br>Longitude and latitude of the studied sites, extracted by Google Map
Type of coastal intervention	What type of coastal intervention is discussed in the document?	1 = Soft<br>2 = Hybrid<br>3 = Hard	
Describe the intervention in the text			
Ecosystem types	What is the ecosystem associated with the intervention?	1 = Mangrove<br>2 = Salt marsh<br>3 = Unspecified wetland<br>4 = Seagrass<br>5 = Coral reef<br>6 = Oyster reef<br>7 = Kelp bed<br>8 = Other (River basin, beaches and dunes, catchment)	
Temporal scale	What is the temporal scale of the research?	1 = Cross-sectional study<br>2 = Longitudinal<br>3 = Mixed	
Spatial Scale	What is the geographical scale of the research?	1 = Local<br>2 = Regional<br>3 = Regional<br>4 = Global	
Responding actor	Who is reported as engaging with the intervention	1 = International or multinational governance institution<br>2 = Government (national, sub national, local)<br>3 = Private sector<br>4 = Civil society<br>5 = Local communities<br>6 = Others	
Data collection	What is the method for data collection?	Open text	
Data analysis	What is the method for data analysis?	Open text	
Framework	What is the theoretical framework?	Open text	
Discipline	From what academic discipline the paper belongs to	Open text	
**Data for meta-analysis**			
Climate change risk reduction	Wave attenuation (WA)	1 = Wave height reduction<br>2 = Wave energy reduction<br>3 = Transmission coefficient	Extract mean, sample size, standard errors or standard deviation

Climate change risk reduction	Shoreline response (SR)	1 = Accretion change rate<br>2 = Elevation change rate<br>3 = Sediment accumulation change rate	Extract mean, sample size, standard errors or standard deviation
Climate change mitigation capacity	Carbon storage (Carbon)	1 = Aboveground biomass<br>2 = Foliage pool<br>3 = Belowground biomass<br>4 = Fine root pool<br>5 = Stump pool<br>6 = Soil C uptake<br>7 = Gross primary production<br>8 = Carbon sequestration rate<br>9 = Other C uptake measure	Extract mean, sample size, standard errors or standard deviation
*to avoid double counting, if studies report both total C uptake and individual partly C uptake (e.g. aboveground, underground biomass), we only extracted the value for total C uptake			
Climate change mitigation capacity	GHG emission (GHG)	1 = CO_2 emission<br>2 = N_2O emission<br>3 = CH_4 emission<br>4 = Ecosystem respiration<br>5 = Other carbon source	Extract mean, sample size, standard errors or standard deviation
Cost-benefit analysis	Cost types	1 = Total cost<br>2 = Construction cost<br>3 = Initial investment cost<br>4 = Operational cost<br>5 = Maintenance cost<br>6 = Utility and contract<br>7 = Labour cost<br>8 = Land purchase<br>9 = Opportunity cost<br>10 = Plantation cost<br>11 = Repositories/ compensation for resource damage	
Cost-benefit analysis	Benefit types	1 = Total benefit<br>2 = Avoided damage cost<br>3 = Fisheries<br>4 = Timber production<br>5 = Raw material provision<br>6 = Climate regulating<br>7 = Carbon sequestration<br>8 = Water purification<br>9 = Coastal protection<br>10 = Flood control<br>11 = Biodiversity<br>12 = N and C removal<br>13 = Education and research<br>14 = Tourism and recreation<br>15 = Total direct use value<br>16 = Total indirect use value<br>17 = Optional value	
Other factors	Year of project<br>Species name<br>Characteristics of intervention<br>Variable name<br>Unit of variable<br>Data collection method		

Supplementary Table 2. Quality criteria for reviewed studies. Source ref[1]

**Quality checklist question**	**Score** Yes=1, No=0
INTERNAL VALIDITY	
Research aim	
1. Does the study address a clearly focused question?	
2. Does the question match the answer?	
Data collection	
3. Was the population/area of interest defined in space, time and size?	
4. Selection bias: Was the sample area representative for the population defined?	
5. Was the sample size appropriate?	
6. Was probability/random sampling used for constructing the sample?	
7. If secondary data were used, did an evaluation of the original data take place?	
8. If data collection took place in form of a questionnaire, was it pre-tested/piloted?	
9. Were the data collection methods described in sufficient detail to permit replication?	
Analysis	
10. Were the statistical/analytical methods described in sufficient detail to permit replication?	
11. Is the choice of statistical/analytical methods appropriate and/or justified?	
12. Was uncertainty assessed and reported?	
Results and Conclusions	
13. Do the data support the outcome?	
14. Magnitude of effect: Is the effect large, significant and/or without large uncertainty?	
15. Are all variables and statistical measures reported?	
16. Attrition bias: Are non-response/drop-outs given and is their impact discussed?	
DESIGN-SPECIFIC ASPECTS	
Review	
17. Is there a low probability of publication bias?	
18. Is the review based on several strong-evidence individual studies?	
19. Do the studies included respond to the same question?	
20. Are results between individual studies consistent and homogeneous?	
21. Was the literature searched in a systematic and comprehensive way?	
22. Was a meta-analysis included?	
23. Were appropriate a priori study inclusion/exclusion criteria defined?	
24. Did at least two people select studies and extract data?	
FOCUS-SPECIFIC ASPECTS	
Quantification	
25. Is the unit of the quantification measurement appropriate?	
26. Was temporal change (e.g. annual or long-term) of quantities measured (e.g. species abundance or an ecosystem service) discussed?	
Valuation	
27. If discounting of future costs and outcomes is necessary, was it performed correctly?	
28. If aggregate economic values for a population were estimated, was this estimation consistent with the sampling and the definition of the population?	

Note: For each criterion answered with a 'yes' the study receives one point, else it receives zero points. If a question is not applicable for the specific study it may be left out, especially for design-specific and focus-specific aspects. Based on the aggregate score studies are characterized as having (a) Weak evidence if score is <24%; (b) Moderate evidence if score is 25%-49%; (c) Strong evidence if score is 50%-74%; and (d) Very strong evidence if score >75%.

Supplementary Table 3: Definition of terminology in this review

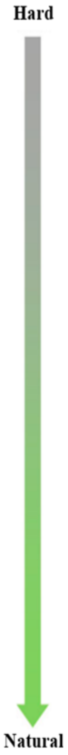

Type of coastal defence	Definition
Hard measures	Hard engineering options are structures used to protect coastal communities from natural disasters such as seawalls, dykes, breakwaters, and levees.
Hybrid measures	Hybrid measures refer to a combination of hard engineering structures integrated with restored or created coastal habitats such as breakwaters in front of salt marshes and rock sills with oyster reefs. Environmental-friendly engineering structures such as vegetated engineering or bamboo fences is also included in this category.
Soft measures	Soft measures refer to the restoration, rehabilitation, reforestation, plantation, protection and management of natural environment such as mangroves, salt marshes, coral reefs, seagrasses, kelp beds, beaches and sand dunes for coastal defences[9]<br>- Restoration and rehabilitation: interventions to restore natural or semi-natural ecosystems including ecological intervention, functional restoration, revegetation, and habitat restoration<br>- Reforestation and plantation: interventions to create or newly plant an ecosystems type in a place of naturally existing ones (reforest formerly degraded saltmarshes) or in a place where natural habitats are modified (e.g. introduction of exotic species to reforest degraded forests)<br>- Protection and management: Interventions such as protected areas, specific site conservation measures, reserved areas, and locally managed areas.<br>Within the scope of this paper, soft measures do not include non-nature-based solutions such as flood early-warning systems and evacuation systems.
Natural measures	Natural coastal habitats generally include undisturbed, naturally regenerated, or degraded natural habitats that lie between coastal communities and the coastlines and provide protection against natural hazards. These natural habitats, affected or not affected by human activities, do not entail intentional human intervention for active restoration.
Unvegetated natural systems	Unvegetated systems refer to unvegetated sand flats, mud flats, open beaches, or abandoned aquaculture ponds.

Note: Among the measures included in this Table, hybrid, soft and natural measures fall under the umbrella of Nature-based solutions (NbS)

Supplementary Table 4: Keywords used to identify the reviewed literature

Search terms groups	Specific search terms
Layer 1<br>(a) Nature-based solution	**TS**= ("ecosystem approach*" OR "ecosystem-based" OR "community-based" OR "disaster risk reduction*" OR "building with nature" OR (natur* AND near/1 (solution* OR approach*)) OR (infrastructure AND n ear/1 (green OR natural OR blue OR ecological)) OR (integrated AND n ear/2 AND management) OR "natural resource* management" OR mangrove* OR marsh* OR wetland* OR "coral reef*" OR "oyster reef*" OR seagrass* OR kelp*<br><br>**AND**<br><br>**TS**=protect* OR manage* OR restor* OR replant* OR rehabilitat* OR reforest* OR plant* OR recover* OR afforest* OR regener* OR creat* OR hybrid)<br><br>**AND**<br><br>**TS** = beach* OR “sand dune*” **AND TS**=(nourish* OR plant* OR advance* OR artificial OR creat* OR regener* OR creat* OR hybrid OR recover)
(b) Hard engineering measures	**TS**=breakwater* OR groyne* OR groin* OR revetment OR seawall* OR riprap* OR "hard defence*" OR "engineer* solution*" OR "engineer*" OR "eco-engineer*" OR "ecological engineer*"
Layer 2: Coastal defence and adaptations	TS=((coast* OR shore* OR marine*) AND near/1 (protect* OR defense* OR defence* OR manage* OR restor* OR adapt*)) OR "hazard mitigat*" OR "hazard reduc*" OR "risk* reduc*" OR "risk* mitigat*" OR "adaptation*"
Layer 3:<br>(a) Wave attenuation and shoreline response	**TS=** ("wave height" OR "wave energy" OR "wave attenuate*" OR "wave breaking" OR "shore* stabili*" OR "beach* stabili*" OR accretion OR erosion OR "elevation change* OR "sediment* accumulation")
(b) carbon storage and GHG emissions	**TS**= ("carbon sequestration" OR "carbon stor*" OR "biomass" OR "carbon source*" OR GHG OR "greenhouse gas*" OR "carbon emission*" OR "carbon flux*" OR "carbon sink")
(c) cost-benefit	**TS = (**economic* OR valu* OR monetary OR valuat* OR benefit* OR cost*)
**Search term sequence:**<br>- Group 1 terms: 1a AND 2 AND 3a (Coastal NbS and risk reduction functions)<br>- Group 2 terms: 1a AND 2 AND 3b (Coastal hard measures and risk reduction functions)<br>- Group 3 terms: 1a AND 2 AND 3c (Coastal NbS and mitigation functions)<br>- Group 4 terms: 1b AND 2 AND 3a (Coastal hard measures and mitigation functions)<br>- Group 5 terms: 1b AND 2 AND 3b (Coastal NbS and cost-effectiveness)<br>- Group 6 terms: 1b AND 2 AND 3c (Coastal hard measures and cost effectiveness)	

Supplementary Table 5: Countries represented in the study sites in the reviewed papers

Country	WA	SR	MC	CBA	Total
**Asia**					93
Bangladesh		1		3	4
China	8	3	9	7	27
India	1	2	4	3	10
Indonesia	3		3	7	13
Iraq				1	1
Japan				2	2
Jawa Barat	1				1
Korea	2				2
Malaysia				3	3
Madives	2				2
Nepal				2	2
Philippines			3	5	8
Sri Lanka		3			3
Taiwan	1			3	4
Thailand			1	3	4
Vietnam			4	2	6
**Europe**					35
Belgium		2			2
Bosnia and Herzegovina				1	1
Denmark				1	1
Finland				1	1
France	1			2	3
Germany			1	1	2
Hungary				1	1
Iberian Penisula		1	1		2
Italy	1	2	3	4	10

Netherlands	1				1
Slovakia				1	1
Spain		1	1	2	4
Portugal		1		1	2
United Kingdom	3	3	2	1	9
**North America**					96
Canada		2	3	1	6
Caymon Island	1	1			1
Costa Rica				1	1
Dominica				1	1
Guam	1			1	2
Gulf of Mexico				1	1
United States	25	26	17	23	91
Nova Scotia				1	1
Quebec			1		1
**Oceania**					28
Australia	14	2	1	6	23
Marshall Islands	1				1
Mayotte Islands	1				1
New Zealand	1			1	2
Vanuatu				1	1
**South America**					4
Brazil				3	3
Ecuador				1	1
**Africa**					6
Egypt		2			2
Ghana				1	1
Kenya				2	2
Tazania				1	1

Note: WA is wave attenuation functions, SR is shoreline response functions, MC is mitigation capacity, BCA is benefit cost analysis

Supplementary Table 6: Two-sided Cochran's Q statistics test for different functions

	Function	Cochran's Q	df (=n-1)	P-value
Soft vs. unvegetated natural systems	Wave attenuation	564.10	24	<0.001
	Accretion change rate	625.98	23	<0.001
	Elevation change rate	236.10	11	<0.001
	Sediment accumulation change rate	123.65	7	0.0087
	Shoreline response	991.11	45	<0.001
	**Risk reduction function**	1553.30	68	<0.001
	Carbon storage	349.13	37	<0.001
	GHG emissions	98.91	9	<0.001
	**Mitigation function**	717.36	56	<0.001
	Total outcome	2159.88	125	<0.001
Soft vs. natural	Wave attenuation	0.04	2	0.8483
	Accretion change rate	328.02	25	<0.001
	Elevation change rate	469.00	18	<0.001
	Sediment accumulation change rate	196.64	13	<0.001
	Shoreline response	1046.11	58	<0.001
	**Risk reduction function**	1047.36	60	<0.001
	Carbon storage	743.54	99	<0.001
	GHG emissions	96603.40	73	<0.001
	**Mitigation function**	97353.40	174	<0.001
	Total outcome	98802.60	235	<0.001
Hybrid vs. unvegetated natural systems	Wave attenuation	4849.32	31	<0.001
	Accretion change rate	33.95	1	<0.001
	Elevation change rate	76.72	23	<0.001
	Sediment accumulation change rate	-	-	-
	Shoreline response	110.80	25	<0.001
	**Risk reduction function**	5306.71	59	<0.001
	Total outcome	5218.29	56	<0.001
Hybrid vs. natural	Wave attenuation	4286.25	4	-
	Accretion change rate	-	-	-
	Elevation change rate	67.91	12	<0.001
	Sediment accumulation change rate	42.25	9	<0.001
	Shoreline response	116.85	23	<0.001
	**Risk reduction function**	4512.86	28	<0.001
	Carbon storage	74.64	9	<0.001
	GHG emissions	-	-	-
	**Mitigation function**	74.64	9	<0.001
	Total outcome	4589.08	37	<0.001
Hard vs. unvegetated natural systems	Wave attenuation	256.48	19	<0.001
	Accretion change rate	142.50	13	<0.001
	Elevation change rate	24.71	3	<0.001
	Sediment accumulation change rate	8.81	3	0.0320
	Shoreline response	180.40	21	<0.001
	**Risk reduction function**	684.29	40	<0.001
	Total outcome	684.29	40	<0.001
Hard vs. natural	Wave attenuation	51.82	5	<0.001
	Accretion change rate	-	-	-
	Elevation change rate	75.39	5	<0.001
	Sediment accumulation change rate	-	-	-
	Shoreline response	75.39	5	<0.001
	**Risk reduction function**	133.69	11	<0.001
	Total outcome	133.69	11	<0.001

Supplementary Table 7: Adjusted estimates after excluding outliers

	Function	Original estimates			No of	Adjusted estimate		
		SMD	Low	High	outlier	SMD	Low	High
Soft vs. unvegetated natural systems	Wave attenuation	6.02	0.75	11.47	1	5.90	0.76	12.53
	Accretion change rate	1.63	-2.05	5.32	2	-1.58	-6.32	4.56
	Elevation change rate	3.69	1.05	6.34	1	4.54	1.85	7.23
	Sediment accumulation change rate	1.68	0.08	3.27	0	1.68	0.08	3.27
	Shoreline response	1.32	-1.07	3.70	0	1.32	-1.07	3.70
	**Risk reduction function**	3.60	-0.16	7.58	1	2.67	-2.34	7.68
	Carbon storage	5.98	0.50	11.47	2	3.08	1.66	4.50
	GHG emissions	-1.47	-2.21	-0.72	3	-1.09	-1.46	-0.72
	**Mitigation function**	1.18	-0.40	2.76	3	1.14	-0.39	2.67
	Total outcome	2.9425	0.8562	5.0289	2	1.8799	1.0299	4.7896
Soft vs. natural	Wave attenuation	2.17	0.74	3.59	0	2.17	0.74	3.59
	Accretion change rate	2.61	0.55	4.68	2	2.51	0.20	4.81
	Elevation change rate	2.53	0.31	4.74	3	4.99	0.37	9.62
	Sediment accumulation change rate	1.73	-0.57	4.03	0	1.73	-0.57	4.03
	Shoreline response	1.60	-0.11	3.30	5	2.12	-0.36	6.27
	**Risk reduction function**	1.7	0.13	3.34	5	2.66	1.10	6.23
	Carbon storage	-0.13	-0.89	0.63	3	-0.02	-0.53	0.49
	GHG emissions	-0.03	-0.94	0.89	5	-0.42	-1.77	0.92
	**Mitigation function**	-0.32	-0.75	0.12	11	-0.31	-0.90	0.28
	Total outcome	0.25	-0.27	0.78	11	0.51	-0.21	1.24
Hybrid vs. unvegetated natural systems	Wave attenuation	8.34	3.57	13.11	4	9.71	5.80	13.62
	Accretion change rate	0.37	-0.58	1.31	0	0.37	-0.58	1.31
	Elevation change rate	0.54	0.33	0.75	7	0.42	0.15	0.69
	Sediment accumulation change rate	0.93	-0.67	2.52	0	0.93	-0.67	2.52
	Shoreline response	0.54	0.33	0.75	9	0.44	0.17	0.703
	**Risk reduction function**	6.36	2.78	9.94	3	6.66	0.17	0.70
	Carbon storage	0.96	0.96	0.96	0	0.96	0.96	0.96
	**Mitigation function**	0.96	0.96	0.96	0	0.96	0.96	0.96
	Total outcome	5.89	2.50	9.27	3	6.19	3.10	9.27
Hybrid vs. natural	Wave attenuation	5.24	-4.91	15.42	1	0.17	-3.39	3.37
	Accretion change rate	3.13	3.13	3.13	0	3.13	3.13	3.13
	Elevation change rate	-0.15	-3.84	3.55	2	0.45	-0.40	1.30
	Sediment accumulation change rate	3.34	-1.13	7.80	1	1.10	0.05	2.16
	Shoreline response	1.68	-0.83	4.19	2	0.40	-1.41	2.21
	**Risk reduction function**	2.66	-0.45	5.76	2	2.71	-0.24	5.66
	Carbon storage	-1.51	-3.00	0.02	2	-1.51	-3.39	0.37
	GHG emissions	-	-	-	-	-	-	
	**Mitigation function**	-1.51	-3.00	0.02	2	-1.511	-3.39	0.37
	Total outcome	1.22	-1.07	3.51	4	0.65	-1.03	2.33
Hard vs. unvegetated Natural systems	Wave attenuation	6.29	2.78	9.80	0	6.29	2.78	9.80
	Accretion change rate	2.55	0.12	4.97	1	4.06	0.94	8.05
	Elevation change rate	10.80	-8.02	29.70	1	0.96	0.05	1.88
	Sediment accumulation change rate	1.37	0.69	2.04	0	1.37	0.69	2.04
	Shoreline response	2.01	0.82	3.20	1	2.87	1.09	4.66
	**Risk reduction function**	3.96	1.91	6.06	0	3.96	1.91	6.06
	Total outcome	3.96	1.91	6.06	0	3.96	1.91	6.06
Hard vs. natural	Wave attenuation	-2.26	-6.43	1.91	1	-0.97	-1.84	-0.09
	Accretion change rate	-	-	-	-	-	-	
	Elevation change rate	-0.03	-3.12	3.06	1	-1.61	-2.56	0.67
	Sediment accumulation change rate	-	-	-				
	Shoreline response	-0.03	-3.13	3.06	1	-1.61	-2.56	0.67
	**Risk reduction function**	-2.26	-6.43	1.91	1	2.87	1.09	4.66
	Total outcome	-2.26	-6.43	1.91	1	2.87	1.09	4.66

Note: Outliers are identified using Cook's Distance. Lines in red indicate removing outliers has significant effect on the results.

Supplementary Table 8: Adjusted estimates after excluding functions with small number of observations (n<3)

Comparison	Aggregate Function	Figure	Original estimates			Removed individual functions	Adjusted estimates		
			SMD	Low	High		SMD	Low	High
Soft vs. natural	Risk reduction	Figure 1a	1.7311	0.1266	3.3355	2	1.597	0.1106	3.3045
	Overall performance	Figure 1a	0.2547	-0.2747	0.7842	2	0.2250	-0.3101	0.7601
Hybrid vs. natural	Risk reduction	Figure 1b	2.6576	-0.4482	5.7635	1	2.6209	-0.7830	6.0247
	Overall performance	Figure 1b	1.2212	-1.0676	3.5099	1	1.113	-1.3041	3.5301
Hard vs. natural	Risk reduction	Figure 1c	-2.2604	-6.4284	1.9076	1	-0.345	-2.2292	1.5392
	Overall performance	Figure 1c	-2.2604	-6.4284	1.9076	1	-0.345	-2.2292	1.5392
Hybrid vs. unvegetated	Risk reduction	Figure 1e	6.3589	2.7801	9.9378	2	7.0968	2.8611	11.3325
	Overall performance	Figure 1e	5.8853	2.4971	9.2736	3	7.0968	2.8611	11.3325

Supplementary Table 9: Subset analysis for soft vs. unvegetated tidal flats and bareland

	Outcome	count	study	SMD	Low	High	p-value	se	zval
**Soft/marsh vs unvegetated**	**Overall outcome**	42	19	1.01	-1.50	3.51	0.43	1.28	0.79
	Wave attenuation	14	9	2.86	-1.12	6.84	0.16	2.03	1.41
	Shoreline response	17	7	-1.09	-5.39	3.22	0.62	2.18	-0.49
	carbon storage	4	2	14.08	-9.89	38.04	0.25	12.23	1.151
	GHG emission	7	2	-0.76	-1.46	-0.05	0.03	0.36	-2.11
**Soft/ mangrove vs unvegetated**	**Overall outcome**	57	13	2.12	-0.43	4.67	0.10	1.30	1.63
	Wave attenuation	-	-	-	-	-	-	-	-
	Shoreline response	16	2	4.06	0.72	7.40	0.02	1.70	0.02
	carbon storage	31	7	5.41	-1.74	12.56	0.14	3.65	1.48
	GHG emission	10	4	-1.55	-2.62	-0.48	0.01	0.54	-2.84
**Soft/ wetland vs unvegetated**	**Overall outcome**	3	2	0.24	-3.75	4.24	0.90	2.04	0.12
	Wave attenuation	-	-	-	-	-	-	-	-
	Shoreline response	-	-	-	-	-	-	-	-
	Adaptation capacity	-	-	-	-	-	-	-	-
	carbon storage	1	1	2.33	-	-	-	-	-
	GHG emission	2	1	-1.75	-3.08	-0.42	0.001	0.68	-2.58
	Mitigation capacity								
**Soft/ seagrass/kelp bed vs unvegetated**	**Overall outcome**	9	5	11.80	-4.45	28.04	0.15	8.29	1.42
	Wave attenuation	7	4	14.25	-5.99	34.50	0.17	10.33	1.38
	Shoreline response	-	-	-	-	-	-	-	-
	carbon storage	2	1	2.31	1.86	2.76	0.001	0.23	10.11
	GHG emission	-	-	-	-	-	-	-	-
**Soft/ reef vs unvegetated**	**Overall outcome**	1	1	3.76	-	-	-	-	
	Wave attenuation	1	1	3.76	-	-	-	-	-

	Outcome	count	study	SMD	Low	High	p-value	se	zval
**Soft/ beach and sand dunes vs unvegetated**	**Overall outcome**	14	8	3.99	1.91	6.06	0.0001	1.21	2.71
	Wave attenuation	3	2	6.30	3.96	8.65	0.0001	1.20	5.27
	Shoreline response	11	7	2.87	0.25	5.49	0.0316	1.33	2.15

Supplementary Table 10: Subset analysis of hybrid vs. unvegetated tidal flats and bareland

	Outcome	count	study	SMD	Low	High	p-value	se	zval
**Hybrid/marsh vs unvegetated**	**Overall outcome**	5	3	5.04	-0.01	10.09	0.0503	2.56	1.99
	Wave attenuation	4	2	7.18	1.78	12.59	0.0093	2.76	2.60
	Shoreline response	-	-	-	-	-	-	-	-
	carbon storage	1	1	0.96	-	-	-	-	-
	GHG emission	-	-	-	-	-	-	-	-
**Hybrid/ mangrove vs unvegetated**	**Overall outcome**	11	3	6.81	-0.27	13.90	0.0593	3.61	1.89
	Wave attenuation	11	3	6.81	-0.27	13.90	0.0593	3.61	1.89
	Shoreline response	-	-	-	-	-	-	-	-
	carbon storage	-	-	-	-	-	-	-	-
	GHG emission	-	-	-	-	-	-	-	-
**Hybrid/ wetland vs unvegetated**	**Overall outcome**	3	2	4.14	-3.38	11.65	0.28	3.83	1.08
	Wave attenuation	1	1	8.03					
	Shoreline response	2	1	0.37	-0.58	1.31	0.45	0.48	0.76
	carbon storage								
	GHG emission								
**Hybrid/ seagrass/kelp bed vs unvegetated**	**Overall outcome**	1	1	52.01	-	-	-	-	-
	Wave attenuation	1	1	52.01	-	-	-	-	-
	Shoreline response	-	-	-	-	-	-	-	-
	carbon storage	-	-	-	-	-	-	-	-
	GHG emission	-	-	-	-	-	-	-	-

Supplementary Table 11: Summary of strengths and weaknesses of the different adaptation options requiring human intervention for the study functions

Functions	Hard measures	Natural measures	Soft measures	Hybrid measures
Wave attenuation	Hard measures such as breakwaters attenuate wave energy by 3 mechanisms: wave reflection, wave breaking and vortex generating. As offshore waves pass through these structures, waves' energy and height are reduced. Hard structures are effective and ready to withstand high waves and storms as long as they are built.	Coastal natural habitats such as mangroves and wetlands interact with waves and tides; via friction forces, they can absorb and dissipate wave energy. There are as effective as hard measures in reducing wave height and wave energy. By attenuating waves, coastal habitats also reduce wave set-up and run-up which are effective in flood control.	Newly planted and restored natural habitats can provide the same functions as natural habitats in wave attenuation. However, restored and planted habitats may take time to reach the growth level to provide such functions.	In hybrid structure, grey components such as dikes and breakwaters can provide immediate wave attenuation, while supporting the establishment of green infrastructure such as saltmarshes that deliver long-term benefits. Hybrid options can harness the strengths and optimise the wave attenuation mechanisms of both soft and hard approaches.
Shoreline stabilisation	Hard measures can control erosion by reducing wave damage at shorelines, providing artificial beaches or preventing the longshore transport of sand. For example, beach nourishment can offer a mechanical replacement of sand to advance the shoreline. Dikes can be used to intercept and divert runoff to avoid erosion.	Coastal natural habitats can reduce coastal erosion by absorbing energy created by ocean currents and retaining sand associated with vegetation. Natural habitats are also effective in enhancing sediment and stabilising the shorelines	Plantation and restoration of coastal habitats can result in initial seaward shift and leverage ecogeomorphic feedback between vegetation, sediment accumulation, and organic matter accretion.	Hybrid options can capitalise on the best characteristics of both hard and soft measures in stabilising the shorelines.
Mitigation function	Not applicable	Coastal wetlands are important carbon sinks.	Coastal wetlands are important carbon sinks. However, the degraded wetlands can release substantial GHGs into the atmosphere. Restoration of such degraded forests can reduce such GHG emissions.	Coastal wetlands are important carbon sink. However, the degraded wetlands can release substantial GHGs into the atmosphere. Restoration of such degraded forests can reduce such GHG emissions.
Cost-effectiveness	Moderate to high.<br>High regular maintenance cost for long-term sustainability.	Very high due to the benefits of ecosystem services. Low investment and maintenance costs for conserving and protecting existing ecosystems.	Mostly high due to added benefits of ecosystem services. Low maintenance cost for long-term.	Mostly high due to added benefits of ecosystem services. Medium maintenance cost for long-term.
Other functions: Biodiversity	Hard measures can degrade or destruct the local natural habitats.	EbAs can enhance restoration and conservation of natural habitats.	EbAs can enhance restoration and conservation of natural habitats.	EbAs can enhance restoration and conservation of natural habitats.

Water quality	Hard measures can degrade water quality due to the construction, organic matter accumulation, and algae growth in close- off estuaries	EbAs can improve water quality and enhance nutrient cycling	EbAs can improve water quality and enhance nutrient cycling	EbAs can improve water quality and enhance nutrient cycling
Ecosystem services	Not applicable	- EbAs can improve fisheries, provide recreational services, and increase health due to leisure activities in nature.<br>- Wetlands can be a breeding place for mosquito that could spread diseases.	- EbAs can improve fisheries, provide recreational services, and increase health due to leisure activities in nature.<br>- Wetlands can be a breeding place for mosquito that could spread diseases.	- EbAs can improve fisheries, provide recreational services, and increase health due to leisure activities in nature.<br>- Wetlands can be a breeding place for mosquito that could spread diseases.
Strengths	- Long experience in design and implementation[2].<br>- - Substantial evidence base about the types, functions, protection mechanisms and capacity, and potential risks of different structures[3].<br>- - Immediate effectiveness after construction, which makes them suitable for high-risk contexts[4].<br>- Possible suitability for areas with limited space[5]	- Possible self-adaptiveness to changing climate and self-recovery after disasters[6].<br>- Provision of multiple co-benefits, including climate change mitigation, human wellbeing and biodiversity conservation[6].<br>- - No investment costs and very high economic benefits[7]	- Possible self-adaptiveness to changing climate and self-recovery after a disaster[6].<br>- Provision of multiple co-benefits, including climate change mitigation, human wellbeing and biodiversity conservation[6].<br>- Restored habitats are more effective in risk reduction with time[4].<br>- - Relatively lower investment cost, high economic benefits	- Flexibility in combining hard and soft measures, which allows for innovative context-specific practices when designing coastal defences[3].<br>- Provision of multiple co-benefits, including climate change mitigation, human wellbeing and biodiversity conservation[6].<br>- Greater confidence in shoreline protection by harnessing the advantages of both hard and soft measure[8].<br>- - Suitable for contexts characterised by low-to-high risk urgency[4].
Weaknesses	- Lack of self-adaptiveness to changing climate.<br>- High environmental risks (e.g. structures damage natural habitats or degrade water quality)[5].<br>- Failure to provide co-benefits associated with climate change mitigation, human wellbeing and biodiversity conservation.<br>- - Possibility of creating perceptions of false safety to local communities[3].	- High vulnerability to the rapid degradation of natural coastal habitats worldwide[9].<br>- Substantial recovery time following degradation by natural or human processes[4].<br>- Insufficient for high-risk coastal zones[4].<br>- Significant space requirement for implementation, which makes them possibly unsuitable for dense urban areas[3].	- Long-time requirement to reach maturity and provide risk reduction benefits equivalent to hard structures[10,11].<br>- Effectiveness is highly depended on ecosystem type (Figure 2) and ecological support of the local environment[12].<br>- Lack of suitability for areas that cannot support ecosystems[3].<br>- Significant space requirement for implementation, which makes them possibly unsuitable for dense urban areas.<br>- Distinct human interactions during implementation[12].	Limited implementation knowledge, expertise, and experiences globally<br>- Substantial effort requirement from different stakeholders (e.g. academics, policy-makers, private sector, local communities) to design the best hybrid measure in a given context[12].<br>- Negative environmental impacts of the grey infrastructure component.<br>- High uncertainty over operational parameters and effectiveness during implementation[4].<br>- Distinct human interactions during implementation[12].

**Opportunities**	- Availability of advanced engineering options to modify and develop existing hard structures for different purposes with improved performance.<br>- Good financial investment outlook in the near future as hard measure are always a priority for coastal defence to climate change[13].<br>- High acceptability as it is the standard approach to coastal adaptation globally.	- Widespread coastal conservation efforts globally.<br>- High awareness of many local communities, policy-makers and private sector in managing and protecting natural habitats.<br>- Ongoing collective international efforts and networks for protecting natural ecosystems to reverse ecosystem degradation[14].<br>- Opportunities to create collaborations with indigenous and local communities to improve community-based resource management[15].<br>- Opportunities for creating synergies to achieve climate resilience, enhance human wellbeing, and protect biodiversity [16].<br>- Alignment of policies with funding support.	- Increased international visibility as 2021-2030 is the United Nation Decade on ecosystem restoration.<br>- Ongoing collective international efforts and networks for restoring natural ecosystems to reverse ecosystem degradation[14].<br>- Financial incentives to integrate EbAs for coastal adaptation due to the high maintenance costs of hard structures<br>- Opportunities for creating synergies to achieve climate resilience, enhance human wellbeing, and protect biodiversity.[16]	- Advanced engineering can inspire innovations in the design and improve the acceptability of hybrid EbAs.<br>- Potential to be globally accepted as a standard approach to coastal defence.<br>- Opportunities for creating synergies to achieve climate resilience, enhance human wellbeing, and protect biodiversity.<br>- Alignment of policies with funding support.<br>- Potential of creating interdisciplinary and trans-disciplinary approaches to normalise the application of hybrid EbAs[4].
**Threats**	- Technological limits of the built structures[17].<br>- Possibility of massive failures of built structures due to inappropriate design, construction, maintenance and operation[18].<br>- Financial constraints posed by funding availability[17]<br>- Institutional limits linked to inadequate governance, limited institutional capacity, lack of political will, and existing laws and procedures[17].	- Social/cultural limits to resource management, low local capacity, and difficulty in engaging different stakeholders, education, social beliefs, and worldviews[17].<br>- Institutional limits: inadequate governance, limited institutional capacity, lack of political will, existing laws and procedures.<br>- Biological limits: unsuitable environmental and ecological conditions for ecosystem growth.	- Social/cultural limits to resource management limited local capacity, difficulty in integrating different stakeholders, education, social beliefs, and worldviews.<br>- Institutional limits: inadequate governance, limited institutional capacity, lack of political wills, existing laws and procedures.<br>- Biological limits: unsuitable environmental and ecological conditions for ecosystem growth.	- Technological limits of the built structures.<br>- Improper design, construction, maintenance and operation can lead to massive failures of built structures.<br>- Financial limits of funding<br>- Social/cultural limits to resource management limited local capacity, difficulty in integrating different stakeholders, education, social beliefs, and worldviews.<br>- Institutional limits: inadequate governance, limited institutional capacity, lack of political wills, existing laws and procedures.<br>- Biological limits: unsuitable environmental and ecological conditions for ecosystem growth.

Supplementary Table 12: Benefit-cost ratios for coastal defence options that entail human intervention

	Soft measures (55 projects)	Hybrid measures (19 projects)	Hard measures (24 projects)
**BCR (-2%, 20 years) mean (min, max)**	11.08 (0.23-215.90)	3.17 (0.30-33.40)	3.4 (0.30-49.90)
**BCR (4.5%, 20 years) mean (min, max)**	6.4 (0.10-126.00)	4.2 (0.17-19.50)	4.1 (0.20-33.40)
**BCR (8%, 20 years) mean (min, max)**	11.08 (0.06-95.20)	7.18 (0.12-27.60)	6.14 (0.17-27.60)

LIST OF BOXES

Supplementary Box 1: Sensitivity Analysis

For the sensitivity analysis we identified all possible outliers that may have a significant effect on the pooled effect size using Cook's distance (see Methods). After removing these outliers, we recalculated the pooled effect sizes for all functions (Table S7, Supplementary Material). The differences in effect sizes before and after removing the outliers are rather minor in terms of the direction of effect sizes, the magnitudes and their 95% CIs. We are therefore confident that our results are robust.

Temporal change analysis

For temporal change, the regression tests (see Methods) show no significant correlations between the publication year and the effect sizes for overall outcomes, risk reduction functions, and climate change mitigation functions across most comparison groups (Figure S5, Supplementary Materials). For some individual functions such as wave attenuation between "hybrid vs. unvegetated natural systems" and carbon storage between "soft vs. natural", the effect sizes show significant correlations with the publication year. However, as the estimates are small-scale (3 out of 34 tests showing significant correlations), the temporal change tests suggest that our results are robust.

Publication bias

For publication bias, the Egger test and funnel plots (see Methods) indicate possible publication biases related for the "hybrid vs. unvegetated natural systems" and "hard vs. unvegetated natural systems" comparisons for the wave attenuation function, and the "hard vs. natural" for the shoreline response functions (Figure S6, Supplementary Material). The asymmetry of the funnel plots in the above observations indicates publication bias possibly due to failure to publish certain findings, selective reporting of results, influence of significant effect sizes in the visibility of published studies, and/or sampling biases. Overall, these estimates of publication bias are arguably small-scale in our meta-data analysis, and we can infer that the results are robust. Nevertheless, sampling bias and publication bias are unavoidable in these types of empirical studies. However, by pointing out such potential we encourage the cautious interpretation of these specific results.

Functions with few observations (n<3)

To ensure the robustness of the results, we re-calculated all relevant aggregate functions omitting individual functions with few observations (n<3) (see Table S8, Supplementary Materials). The differences in effect sizes before and after removing the functions with few observations are rather minor in terms of the direction of effect sizes, the magnitudes and their 95% CIs. We therefore are confident that the results are robust.

Supplementary Box 2: Meta-analysis of the grey literature

Literature search and inclusion:

Relevant documents were identified in the BASE database. These documents generally reported the performance of hard structures (e.g. breakwater, seawall, dike, groins) for wave attenuation and shoreline response functions. After full-text screening, 13 documents were deemed relevant for the meta-analysis: 4 were conference papers that were included in the main meta-analysis and 9 were consultancy reports, governmental reports and reports to funders. Due to the quality concerns discussed in the Methods, below we provide the results from a meta-analysis of the nine reports. The reports are mentioned at the bottom of this box.

Results:

Wave attenuation: In total we extracted 26 observations of pairwise comparison between hard structures and unvegetated natural systems for the wave attenuation function. Similar to the meta-analysis described in the Methods, we calculated the effect sizes and used multivariate models which account for non-independence within individual studies to calculate the pool effect size. For the wave attenuation function, the result shows a much higher performance of hard structures compared to unvegetated natural systems (SMD=19.52, 95%CIs = 6.16-33.44, n=26).

Shoreline response: We did not find any pairwise experiment for the accretion rate, elevation change rate, and sedimentation accumulation rate between hard measures and other measures in the grey literature. Hence, while it is not possible to conduct a proper meta-analysis for this function, we provide some descriptive results. Most grey literature reports the substantial effectiveness of hard structures for shoreline stabilisation after construction. Shortly after construction (within 5 years), most of the projects show accretion and sediment accumulation in the shoreline near the structures (see Hashim et al., 2010). For beach nourishment projects with combined hard structures, the volume of sand loss is much lower compared to only beach nourishment after project completion (see Nester, 1982). Beach profiles after hard structure construction show variation in elevation and accretion/erosion rates, which means that accretion is found in some areas and erosion found in other areas (see Hashim et al., 2010, Rosati, 1989).

Carbon emission: We did not find pairwise experiment on GHG emissions between hard measures and other measures. The grey literature reports GHG emission of breakwater construction projects in every phase of the project, from material extraction, to transport, construction, operation/maintenance and disposal. For example, for materials, granite rock extraction can emit 6-781 kgCO_2/tonne whereas for limestone extraction 17 kgCO_2/tonne is given. Concrete production emits 51-112 kgCO_2/tonne. Rubble mound breakwater with concrete amour unit (1.4km in length) is responsible for the emission of 212 million kg CO_2 for material extraction, transport and construction (see Bruce & Chick, 2009).

List of reviewed grey literature:

- Carver, R. Rubble-Mound Breakwater Wave-Attenuation and Stability Tests, Olcott Harbor, New York: Costal Model Investigation. 1991. U.S. Army Engineer Research and Development Center (ERDC) ; Coastal Engineering Research Center (CERC) ; Vicksburg, Mississippi.

- Carver, R. Reef Breakwater Wave-Attenuation and Stability Tests, Burns Waterway Harbor, Indiana. 1995. U.S. Army Engineer Research and Development Center (ERDC) ; Coastal Engineering Research Center (CERC) ; Vicksburg, Mississippi.

- Carver, R. Floating Breakwater Wave-Attenuation Tests for East Bay Marina, Olympia Harbor, Washington: Hydraulic Model Investigation. 1979. U.S. Army Engineer Research and Development Center (ERDC) ; Hydraulics Laboratory (HL) ; Vicksburg, Mississippi.

- Carver, R. Rubble-Mound Breakwater Wave-Attenuation and Stability Test, Burns Waterway Harbor, Indiana. 1993. U.S. Army Corps of Engineers

- Hashim, R et al. Morphological changes in the vicinity of detached breakwater at Sungai Haji Dorani, Peninsular Malaysia. 2010.

- Rosati, J. The Colonial Beach, Virginia, Detaiched Breakwater project. 1989. U.S. Army Engineer Research and Development Center (ERDC) ; Coastal Engineering Research Center (CERC) ; Vicksburg, Mississippi

- Nester, R. Effects of beach nourishment on the nearshore environment in Lake Huron at Lexington Harbor (Michigan), 1982. U.S. Army Engineer Research and Development Center (ERDC) ; Coastal Engineering Research Center (CERC) ; Vicksburg, Mississippi

- Bottin, R. Seabrook Lock Complex, Lake Pontchartrain, LS: Design for Wave Protection at Lock Entrance: Hydraulic Model investigation. 1980. U.S. Army Engineer Research and Development Center (ERDC) ; Hydraulics Laboratory (HL) ; Vicksburg, Mississippi.

- Bruce T. and Chick J. (2009). Energy and Carbon Costing of Breakwaters. Coasts, Marine Structures and Breakwaters 2009, 16–18 September 2009, Edinburgh International Conference Centre, UK. CIRIA (2010).

LIST OF FIGURES

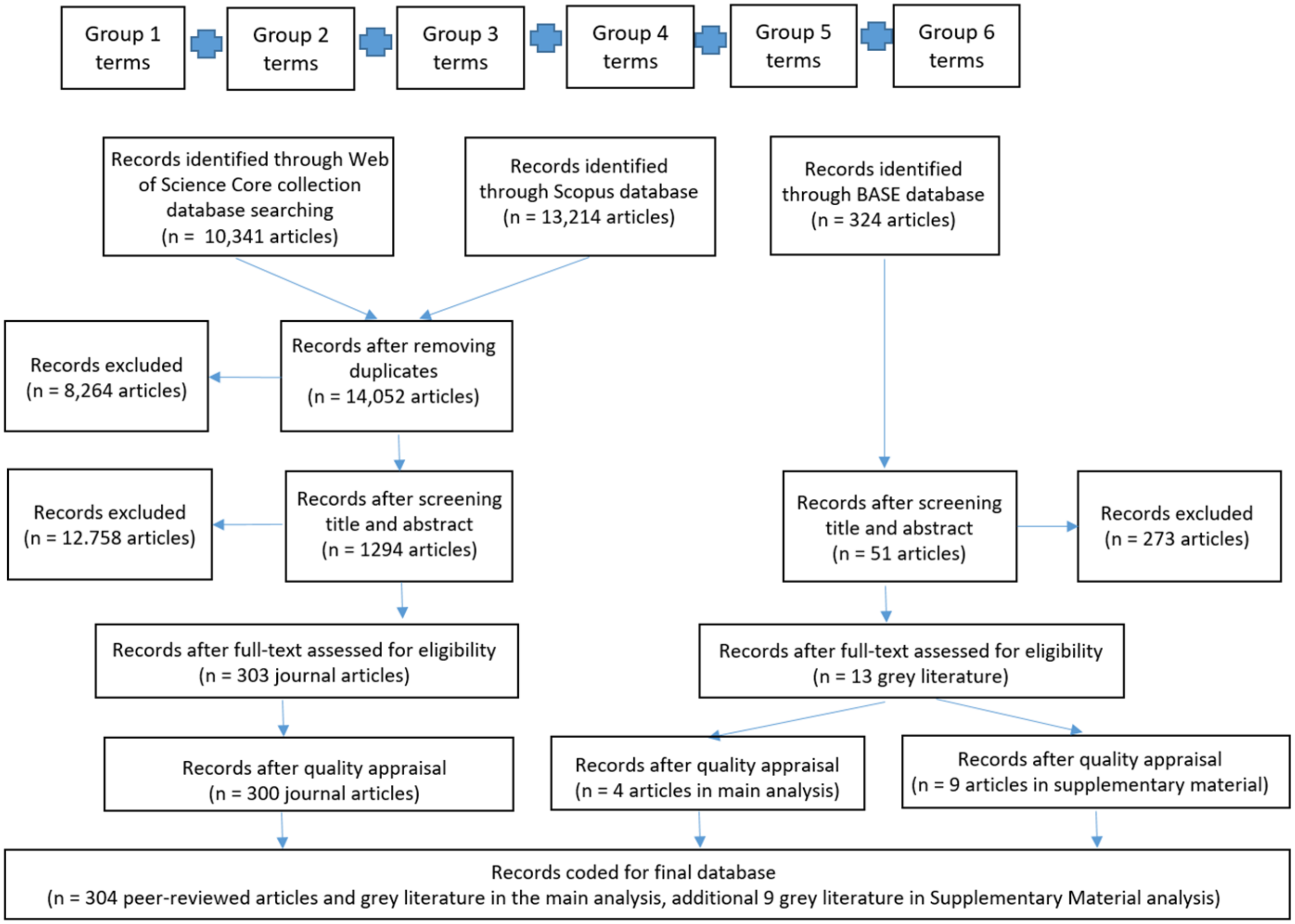

Supplementary Figure 1: PRISMA flow diagram for the study selection and inclusion in the systematic review

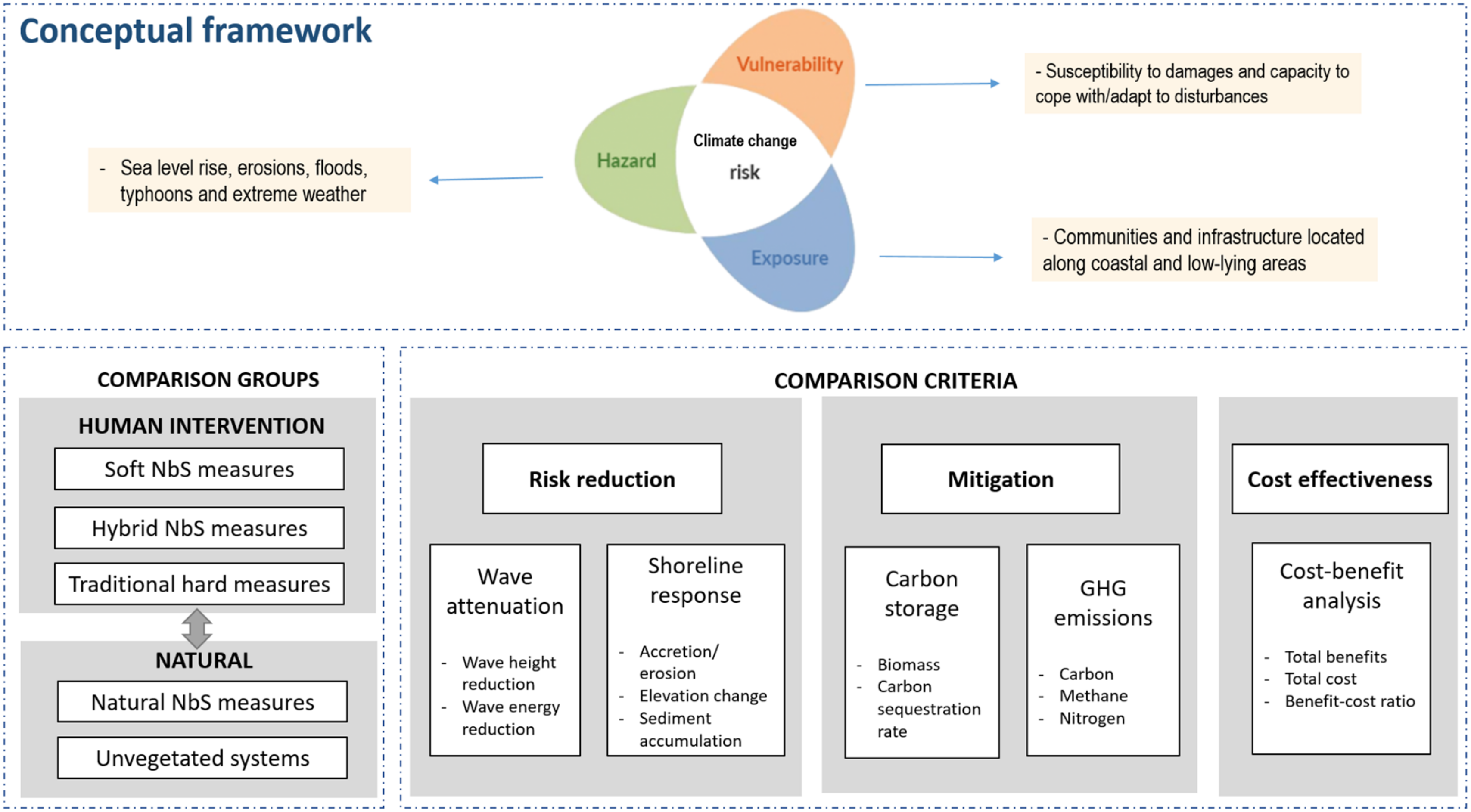

Supplementary Figure 2: Conceptual framework of the meta-analysis

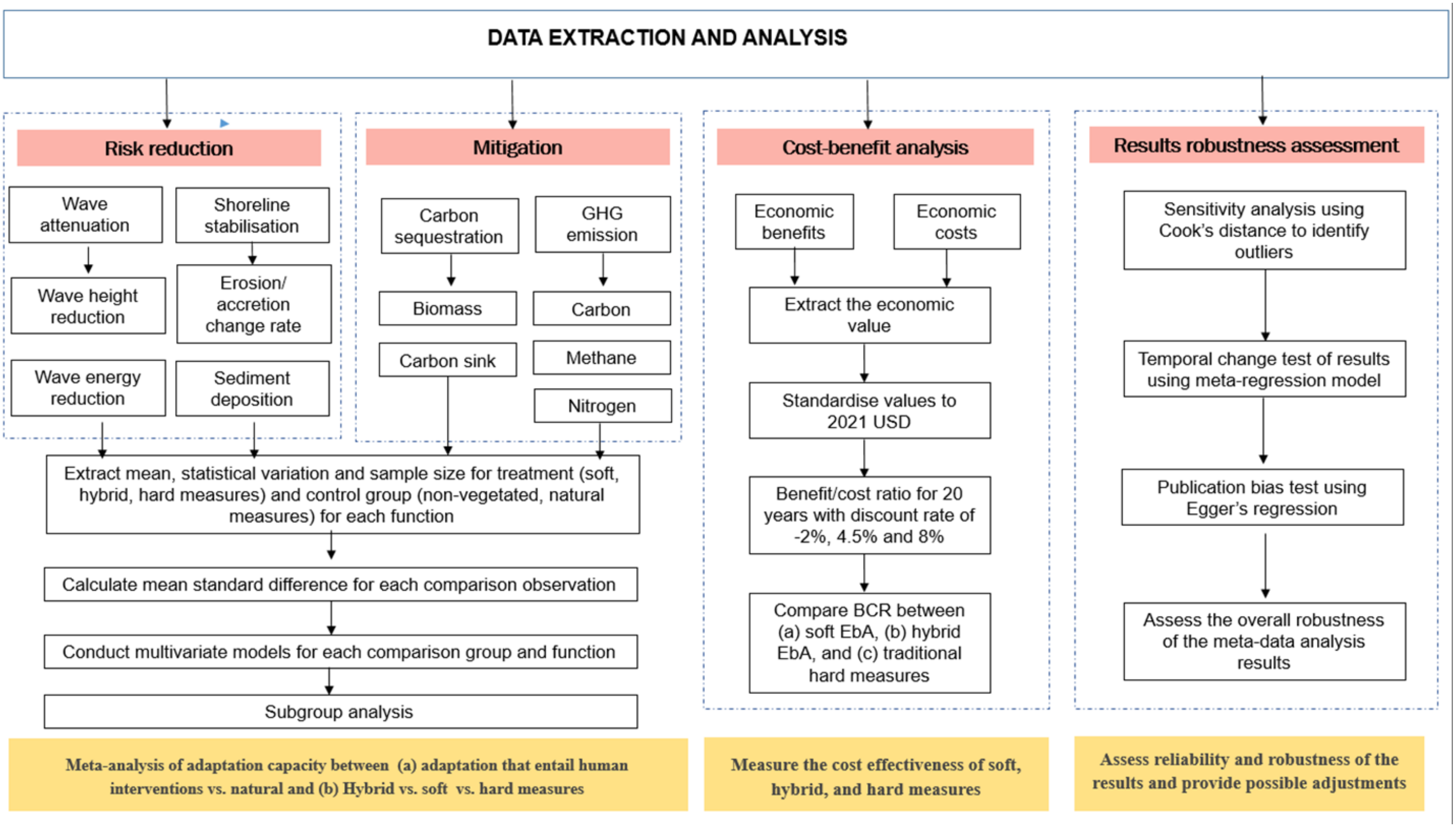

Supplementary Figure 3: Methodological flow of data analysis

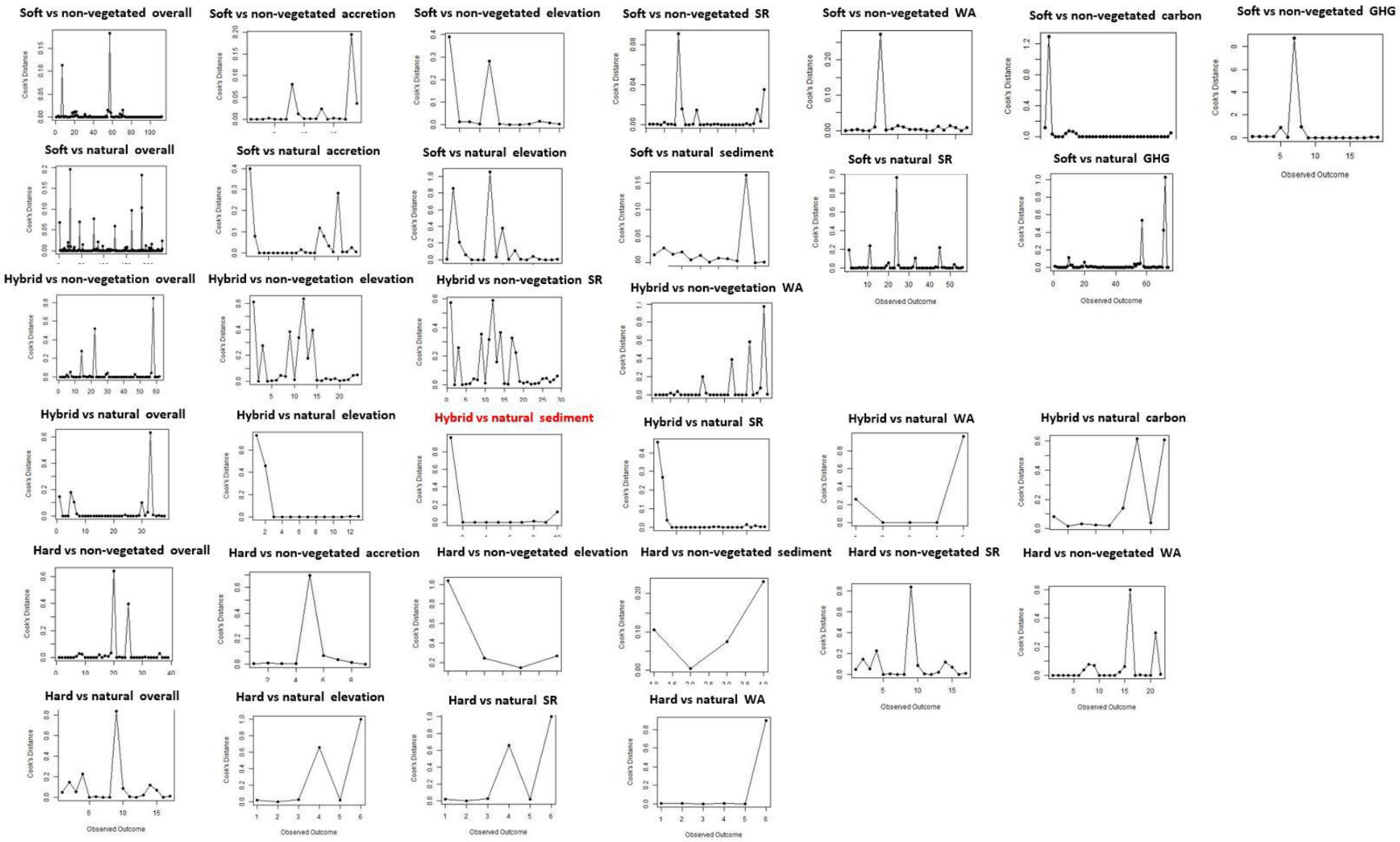

Supplementary Figure 4: Cook's distance diagnoses. Plots detect outliers when a Cook's D is more than the traditional threshold of 4/n (n=sample size). The plots indicated in red text are characterised by significant changes in direction and magnitude of adjusted pool effect sizes when removing the outliers. Refer to Supplementary Table S1 for function abbreviations. Source data are provided as a Source Data file.

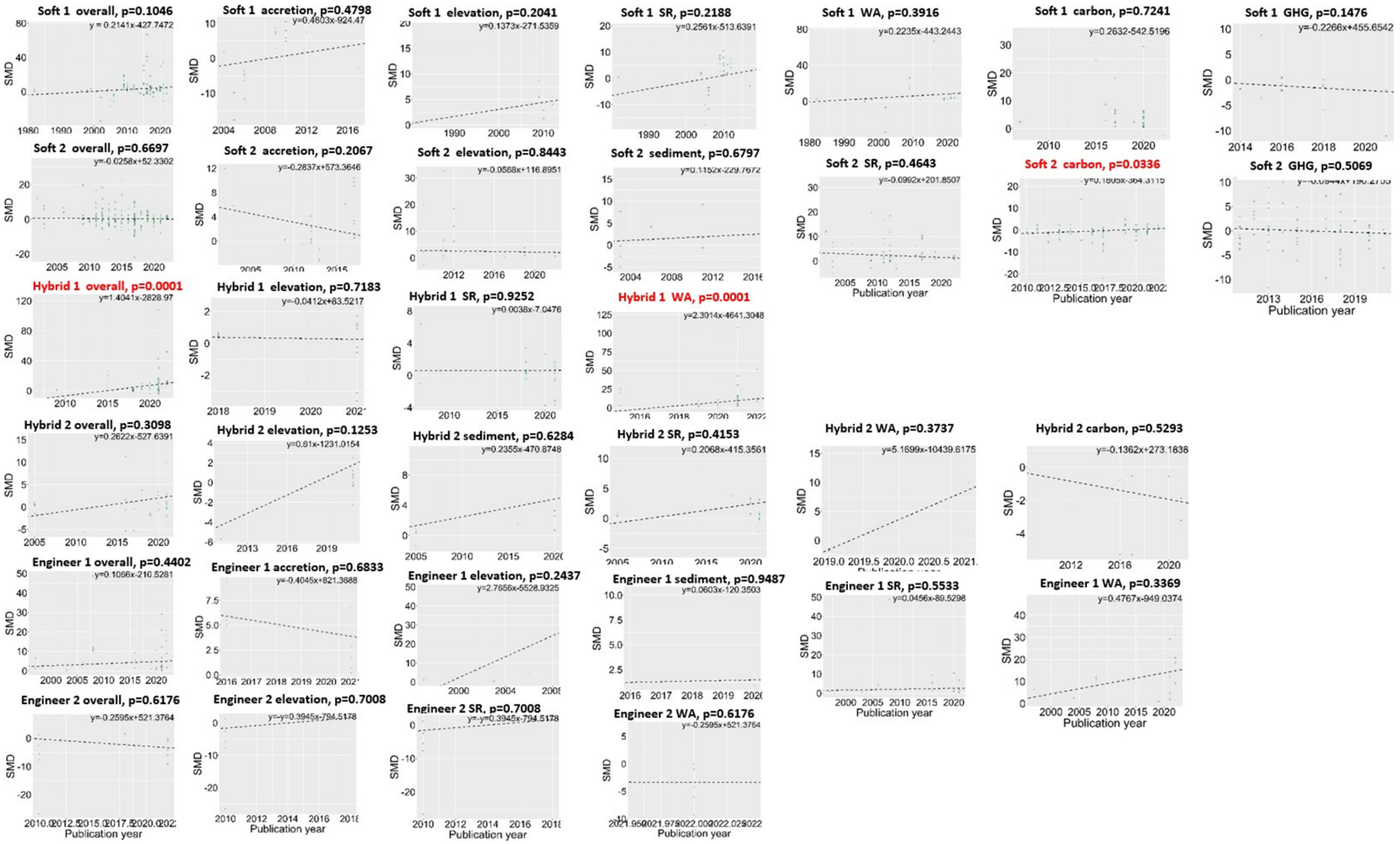

Supplementary Figure 5. Temporal change test. Bubble plots illustrate the meta-regression results between the publication year and the aggregated effect sizes. Dot sizes are in proportion to weight. Black lines indicate the fits for the mixed effects meta-regressions. The value of P is the corresponding p-values of the linear regression model. Refer to Supplementary Table S1 for function abbreviations. The plots indicated in red colour indicates statistical significance. Source data are provided as a Source Data file.

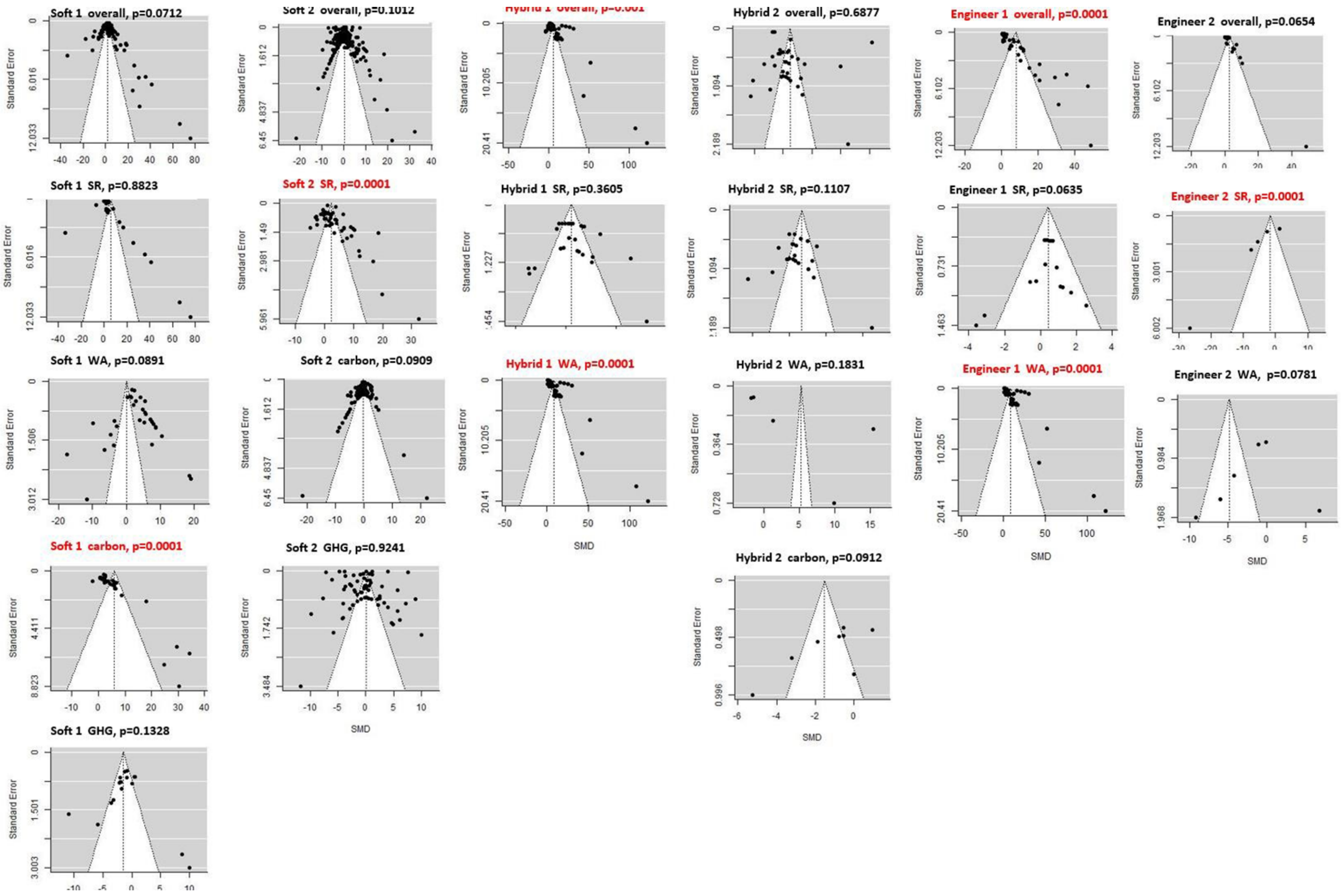

Supplementary Figure 6: Funnel plots of overall adaptation and individual functions. The results of publication bias tests using Egger's regression (z and P-value) are shown in the top of each plots. P<0.05 (indicated in red text) indicates the potential of publication bias. Refer to Supplementary Table S1 for function abbreviations

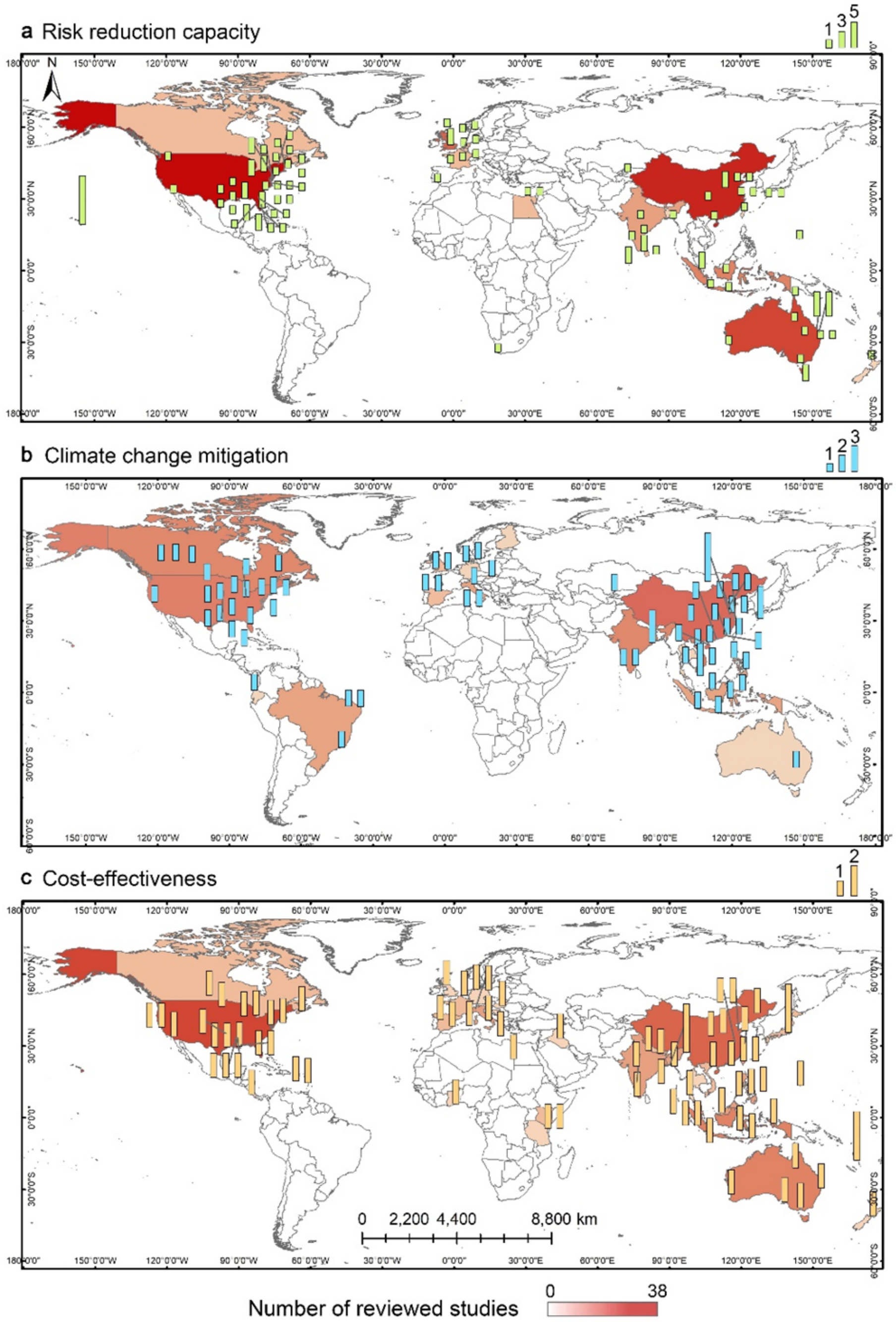

Supplementary Figure 7: Geographical distribution of reviewed studies. Each panel presents the number and location of studies reporting risk reduction (Panel A), climate change mitigation capacity (Panel B), and cost-effectiveness (Panel C). The number of studies is reported both by country (represented by country colour) and by city or province (represented by vertical bar chart). Base map was used under Esri Master License Agreement[19].

Reference list

1. Mupepele, A. C., Walsh, J. C., Sutherland, W. J. & Dormann, C. F. An evidence assessment tool for ecosystem services and conservation studies. *Ecological Applications* vol. 26 1295–1301 (2016).

2. Galili, E. *et al.* A submerged 7000-year-old village and seawall demonstrate earliest known coastal defence against sea-level rise. *PLoS One* **14**, 1–17 (2019).

3. Sutton-Grier, A. E., Wowk, K. & Bamford, H. Future of our coasts: The potential for natural and hybrid infrastructure to enhance the resilience of our coastal communities, economies and ecosystems. *Environ. Sci. Policy* **51**, 137–148 (2015).

4. Morris, R. L., Boxshall, A. & Swearer, S. E. Climate-resilient coasts require diverse defence solutions. *Nat. Clim. Chang.* **10**, 482–490 (2020).

5. Temmerman, S. *et al.* Ecosystem-based coastal defence in the face of global change. *Nature* **504**, 79–83 (2013).

6. Sudmeier-Rieux, K. *et al.* Scientific evidence for ecosystem-based disaster risk reduction. *Nat. Sustain.* **4**, 803–810 (2021).

7. Su, J., Friess, D. A. & Gasparatos, A. A meta-analysis of the ecological and economic outcomes of mangrove restoration. *Nat. Commun.* **12**, (2021).

8. Morris, R. L., Konlechner, T. M., Ghisalberti, M. & Swearer, S. E. From grey to green: Efficacy of eco-engineering solutions for nature-based coastal defence. *Glob. Chang. Biol.* **24**, 1827–1842 (2018).

9. Herbert-Read, J. E. *et al.* A global horizon scan of issues impacting marine and coastal biodiversity conservation. *Nat. Ecol. Evol.* **6**, 1262–1270 (2022).

10. Salmo, S. G., Lovelock, C. & Duke, N. C. Vegetation and soil characteristics as indicators of restoration trajectories in restored mangroves. *Hydrobiologia* **720**, 1–18 (2013).

11. Baumann, M. S. *et al.* Recovery of Salt Marsh Invertebrates Following Habitat Restoration: Implications for Marsh Restoration in the Northern Gulf of Mexico. *Estuaries and Coasts* **43**, 1711–1721 (2020).

12. Anderson, C. C. & Renaud, F. G. A review of public acceptance of nature-based solutions: The 'why', 'when', and 'how' of success for disaster risk reduction measures. *Ambio* **50**, 1552–1573 (2021).

13. Cao, A. *et al.* Future of Asian Deltaic Megacities under sea level rise and land subsidence: current adaptation pathways for Tokyo, Jakarta, Manila, and Ho Chi Minh City. *Curr. Opin. Environ. Sustain.* **50**, 87–97 (2021).

14. Mace, G. M. *et al.* Aiming higher to bend the curve of biodiversity loss. *Nat. Sustain.* **1**, 448–451 (2018).

15. Díaz, S. *et al.* Assessing nature's contributions to people. *Science (80-.).* **359**, 270–272 (2018).

16. Chaigneau, T. *et al.* Reconciling well-being and resilience for sustainable development. *Nat. Sustain.* **5**, 287–293 (2022).

17. Thomas, A. *et al.* Global evidence of constraints and limits to human adaptation Global Adaptation Mapping Initiative Team. *Reg. Environ. Chang.* **21**, 1–15 (2021).

18. Schipper, E. L. F. Maladaptation: When Adaptation to Climate Change Goes Very Wrong. *One Earth* **3**, 409–414 (2020).

19. Esri. Master Agreements; Products and Services Terms of Use. https://www.esri.com/en-us/legal/terms/full-master-agreement (2024).
