# Supplementary material for: Meta-analysis indicates better climate adaptation and mitigation performance of hybrid engineering-natural coastal defence measures
Source: Nat Commun. 2024 Apr 9;15:2870. doi: 10.1038/s41467-024-46970-w (PMC11004181; doi:10.1038/s41467-024-46970-w)
Supplement: Supplementary file 3 — Description of Additional Supplementary Files [file 41467_2024_46970_MOESM3_ESM.pdf]

Description of Additional Supplementary Files

File Name: Supplementary Data 1
Description: This dataset includes the general information of the reviewed studies including study's title, authors, year of publication, location of experiment, types of NbS intervention.

File Name: Supplementary Data 2
Description: this dataset presents the critical appraisal of all reviewed studies.

File Name: Supplementary Data 3.
Description: This dataset provides pair-wise observations between soft vs. unvegetated in academic literature, including data on sample sizes, means, and standard errors of both controlled (unvegetated) and treatment (soft) groups.

File Name: Supplementary Data 4.
Description: This dataset provides pair-wise observations between soft vs. natural in academic literature, including data on sample sizes, means, and standard errors of both controlled (natural) and treatment (soft) groups.

File Name: Supplementary Data 5.
Description: This dataset provides pair-wise observations between hybrid vs. unvegetated in academic literature, including data on sample sizes, means, and standard errors of both controlled (unvegetated) and treatment (hybrid) groups.

File Name: Supplementary Data 6.
Description: This dataset provides pair-wise observations between hybrid vs. natural in academic literature, including data on sample sizes, means, and standard errors of both controlled (natural) and treatment (hybrid) groups.

File Name: Supplementary Data 7.
Description: This dataset provides pair-wise observations between hard vs. unvegetated in academic literature, including data on sample sizes, means, and standard errors of both controlled (unvegetated) and treatment (hard) groups.

File Name: Supplementary Data 8.
Description: This dataset provides pair-wise observations between hard vs. natural in academic literature, including data on sample sizes, means, and standard errors of both controlled (natural) and treatment (hard) groups.

File Name: Supplementary Data 9.
Description: This dataset provides pair-wise observations between hard vs. unvegetated in grey literature, including data on sample sizes, means, and standard errors of both controlled (unvegetated) and treatment (hard) groups.

File Name: Supplementary Data 10.
Description: This dataset includes benefits cost ratios of all hard, hybrid and soft coastal defence projects. The rows highlighted in yellow are outliers and not included in the benefit-cost analysis.
